# Surgical Approaches for Residual Secondary Gaze Diplopia After Strabismus Surgery: A Case Series

**DOI:** 10.7759/cureus.95707

**Published:** 2025-10-29

**Authors:** Juan Lorenzo Simpao, Toshiaki Goseki, Hiromi Onouchi

**Affiliations:** 1 Department of Ophthalmology, International University of Health and Welfare Atami Hospital, Shizuoka, JPN; 2 Department of Ophthalmology, University of Santo Tomas Hospital, Manila, PHL; 3 Department of Ophthalmology, School of Medicine, Kitasato University, Kanagawa, JPN; 4 Department of Ophthalmology, School of Medicine, Tokai University, Kanagawa, JPN

**Keywords:** hess chart, incomitant, residual diplopia, secondary gaze diplopia, strabismus surgery

## Abstract

This case series describes three patients who experienced residual diplopia in secondary gaze positions despite successful strabismus surgery and satisfactory alignment in primary gaze. While initial procedures effectively resolved diplopia in the primary position, symptoms remained in secondary gaze and interfered with daily activities. Each patient underwent an additional surgical procedure specifically addressing the residual diplopia, leading to significant improvement or complete resolution of symptoms. These findings highlight that although primary gaze alignment is the primary goal of strabismus surgery, tailored interventions may be required for patients with symptomatic secondary gaze diplopia to optimize functional outcomes and quality of life.

## Introduction

The management of diplopia generally focuses on correcting misalignment in the primary gaze position. Surgical correction of strabismus is typically aimed at resolving diplopia and misalignment in the primary position. The goal of strabismus surgery is not just cosmetic but reconstructive - restoring normal eye alignment to improve visual function, head posture, binocular fusion, stereopsis, and the binocular visual field. Beyond functional gains, surgery enhances patient well-being, nonverbal communication, and social interactions [1,2]. Diplopia limited to secondary gaze is often considered clinically tolerable and not routinely addressed surgically [3].

However, for certain patients, diplopia in secondary gaze can significantly impair quality of life and functional vision. In such cases, Hess charts are particularly useful for assessing comitant and incomitant deviations across the peripheral field, providing valuable information for surgical planning [4]. For these patients with persistent diplopia in secondary gaze positions that already affect their quality of life, additional strabismus surgery targeting the secondary gaze deviation could be considered an indication for strabismus surgery.

We report a series of cases in which, despite achieving satisfactory primary gaze alignment, patients experienced persistent residual diplopia in secondary gaze that interfered with daily activities. These patients required additional surgical intervention to address the diplopia in secondary gaze, which led to significant symptomatic improvement. This series highlights the potential role of secondary gaze-oriented strabismus surgery in selected cases.

## Case presentation

Methods

This case series includes three adult patients with symptomatic residual diplopia in secondary gaze position after achieving successful strabismus surgery in primary gaze. All patients reported that the residual diplopia in the secondary gaze interfered with their daily activities and affected their quality of life. Each underwent further surgical intervention tailored to their specific motility deficits. Preoperative and postoperative assessments were performed using clinical orthoptic measurements, 9-Gaze application photographs [5], Hess screen chart evaluations, and subjective symptom reports.

Results

Case 1

**Figure 1:**
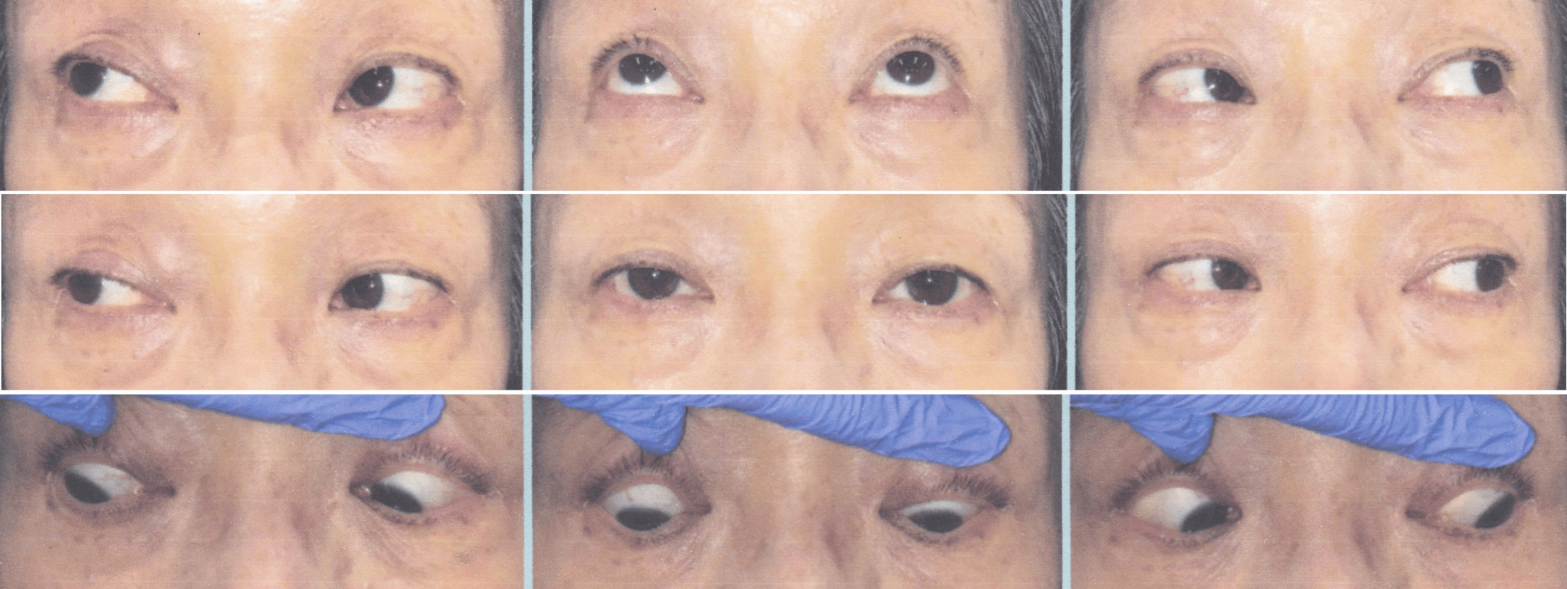
9-gaze photographs of a patient with right superior oblique palsy No obvious ocular motor disorder was observed.

**Figure 2:**
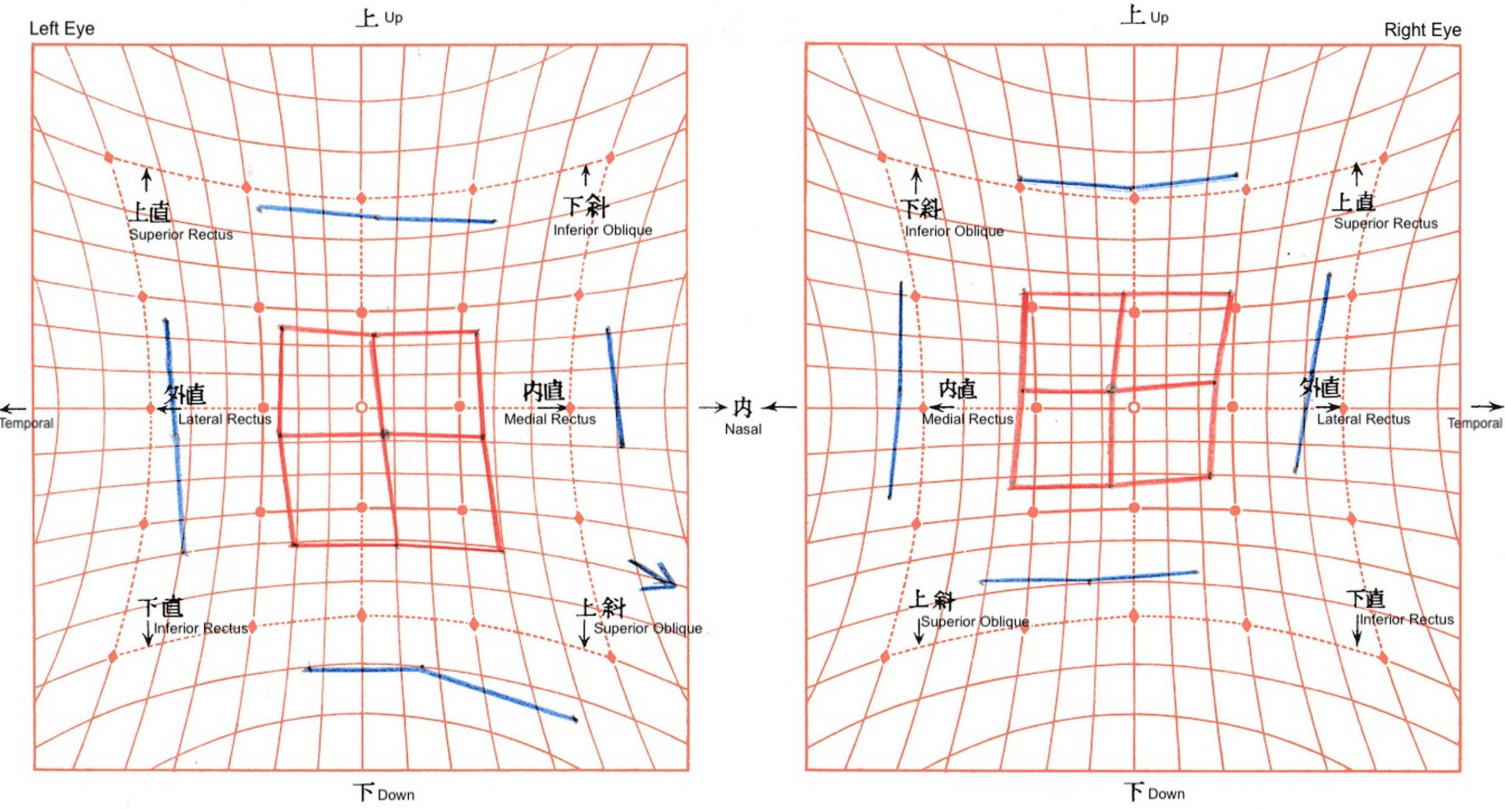
Preoperative Hess screen chart The chart shows left hypodeviation and right hyperdeviation with exodeviation. The left eye demonstrated inferior overaction with loss of comitancy in the inferior gaze position.

A 70-year-old female with a history of right superior oblique palsy with 4 prism diopters (PD) of esotropia, 8 PD of right hypertropia, and 6 degrees of excyclotorsion using cyclophorometer in the primary position. She had no obvious ocular motor disorder, but Hess screen chart showed a few degrees of vertical strabismus (Figures 1, 2).

**Figure 3:**
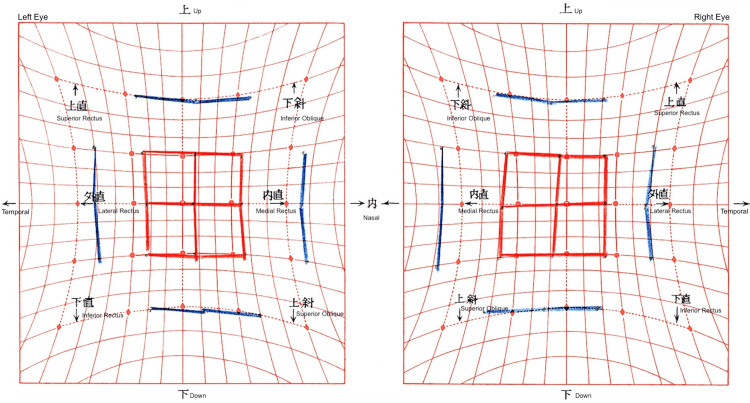
Postoperative Hess screen chart after 2 mm recession with nasal transposition of the left inferior rectus The chart demonstrates marked improvement in alignment, with minimal residual esodeviation but persistent incomitance in lateral gaze.

**Figure 4:**
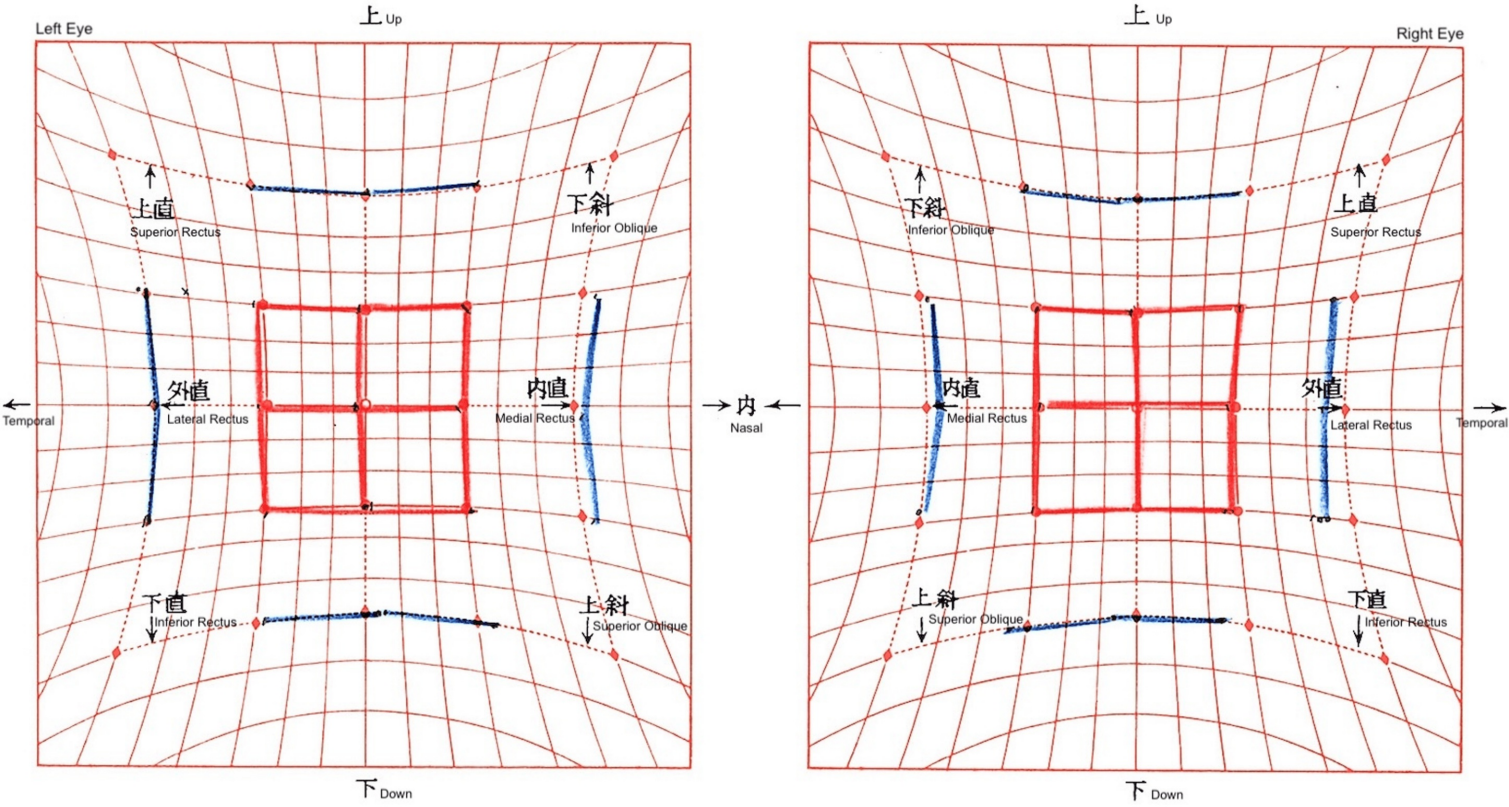
Hess chart after right medial rectus recession The chart demonstrates excellent alignment and resolution of diplopia in secondary gaze one month postoperatively.

She underwent 2 mm recession with half-tendon-width nasal transposition of the left inferior rectus, which improved her diplopia in primary gaze (Figure 3) and corrected her excyclotorsion. However, residual diplopia persisted in lateral gazes (4 PD esotropia), more pronounced on left gaze. A subsequent 4 mm recession of the right medial rectus (MR) resolved diplopia in secondary gaze one month postoperatively (Figure 4).

Case 2

**Figure 5:**
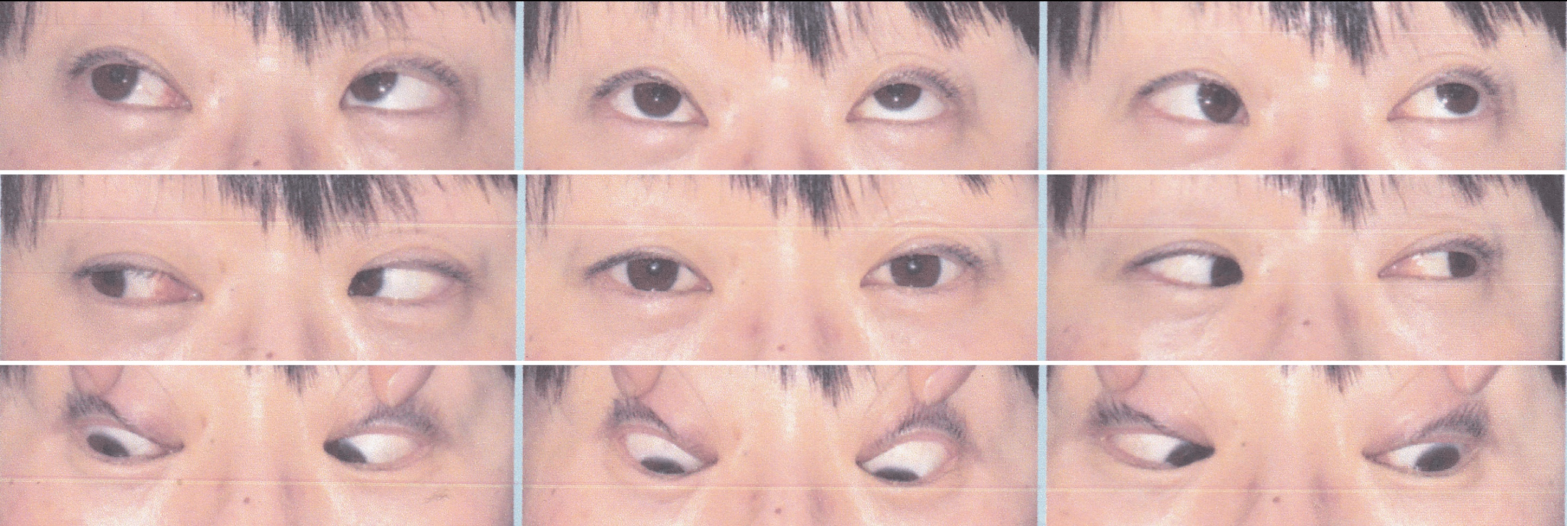
9-gaze photographs of a patient with thyroid eye disease and myasthenia gravis It shows motility restriction in the right eye, particularly in elevation.

**Figure 6:**
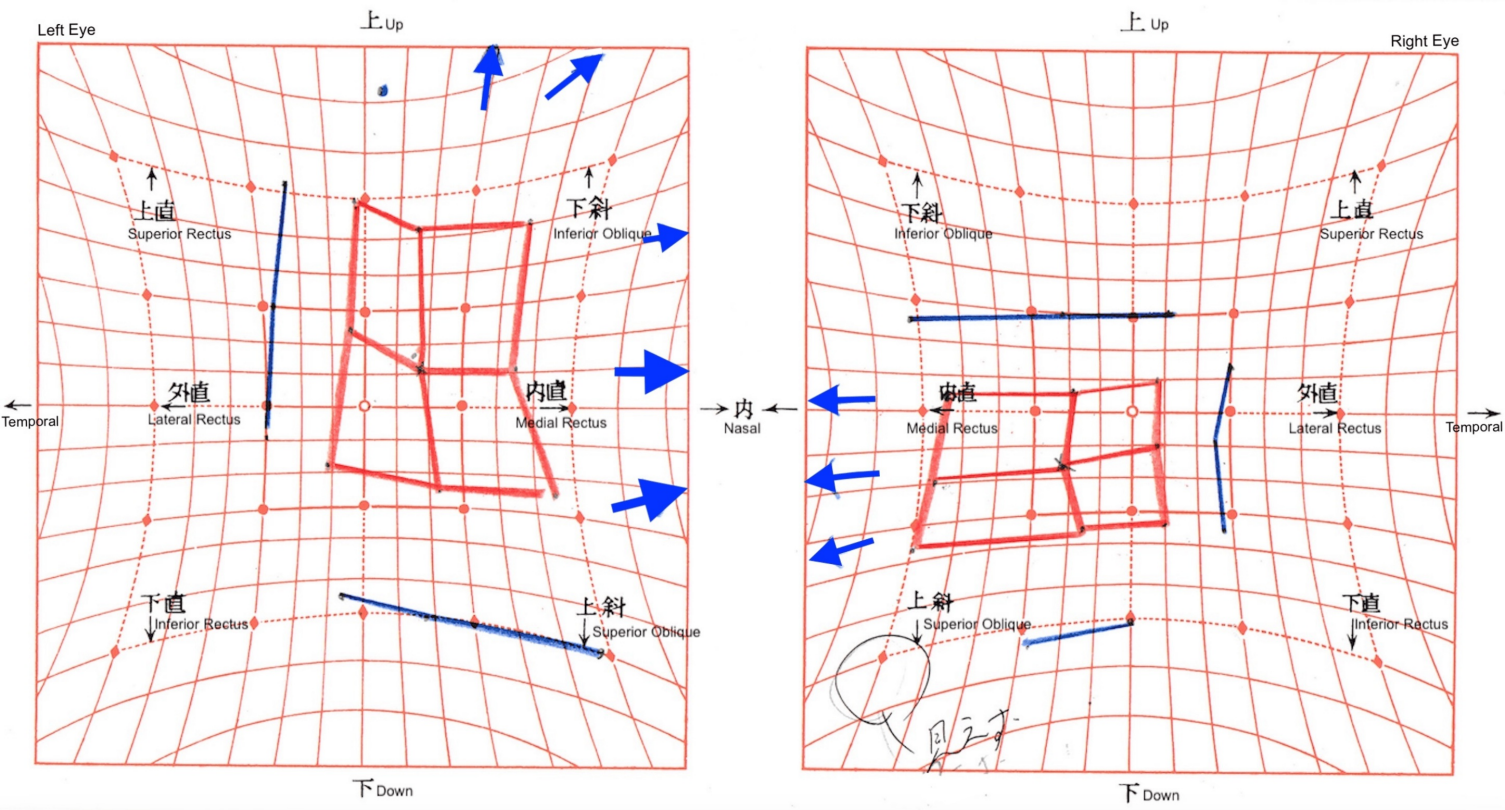
Preoperative Hess screen chart The chart shows right hypodeviation and left hyperdeviation with esodeviation. Restriction of elevation in the right eye resulted in loss of comitancy in upward gaze.

A 57-year-old woman with thyroid eye disease and myasthenia gravis was treated with steroid therapy and orbital decompression. Postoperatively, she developed residual diplopia with 20 PD esotropia, 14 PD right hypotropia, along with limited right eye elevation (Figures 5, 6). 

**Figure 7:**
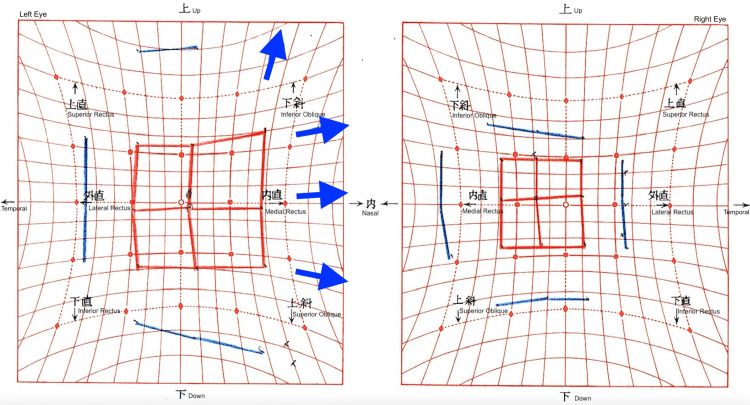
Postoperative Hess screen chart after 4.5 mm inferior rectus recession and 6.5 mm medial rectus recession in the right eye The chart demonstrates marked improvement in alignment, with residual esodeviation and persistent incomitance in right gaze.

**Figure 8:**
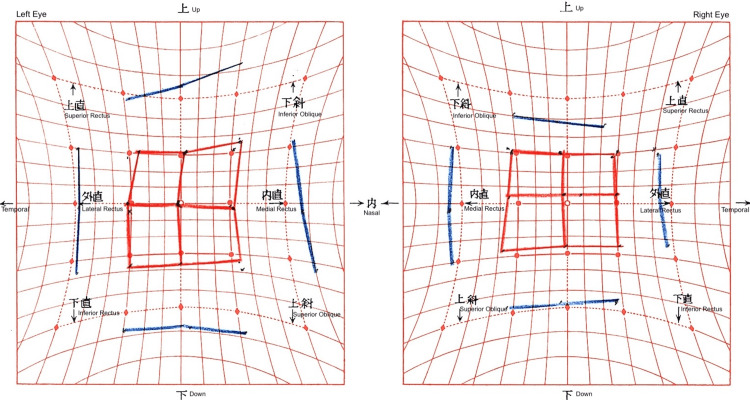
Hess chart after left medial rectus recession The chart demonstrates normal ocular alignment with comitancy in horizontal eye movement and resolution of residual diplopia in right gaze two months postoperatively.

She underwent 4.5 mm inferior rectus recession and 6.5 mm MR recession in the right eye, which resolved diplopia in primary gaze (Figure 7). Residual diplopia in right gaze (10 PD esotropia) was subsequently corrected with a 4.0 mm MR recession in the left eye, resulting in the resolution of diplopia in horizontal eye movement two months postoperatively (Figure 8).

Case 3

**Figure 9:**
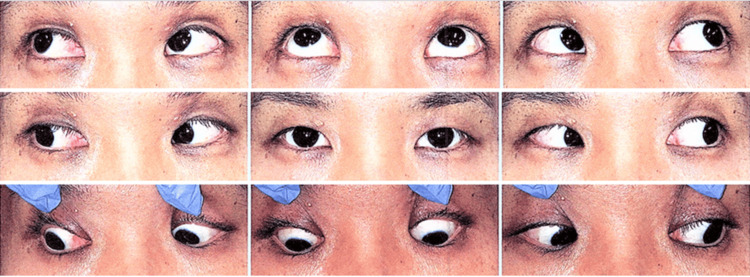
9-gaze photographs of a patient with intermittent exotropia who underwent bilateral rectus recession and right medial rectus recession No obvious ocular motor disorder was observed.

**Figure 10:**
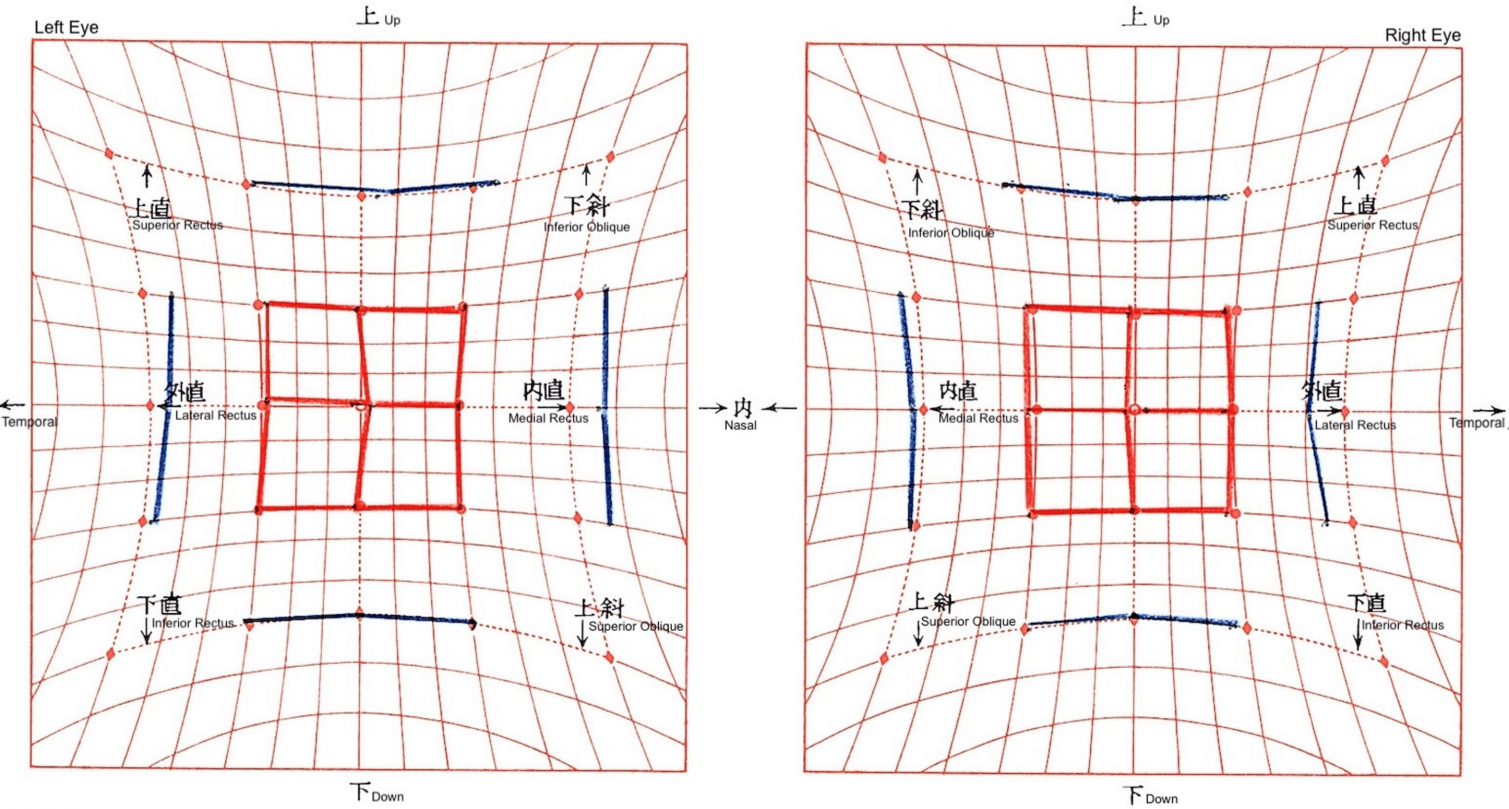
Hess screen chart following bilateral rectus recession and medial rectus recession of the right eye The chart demonstrates normal ocular alignment, with persistent incomitance in right gaze resulting in residual diplopia on right gaze.

**Figure 11:**
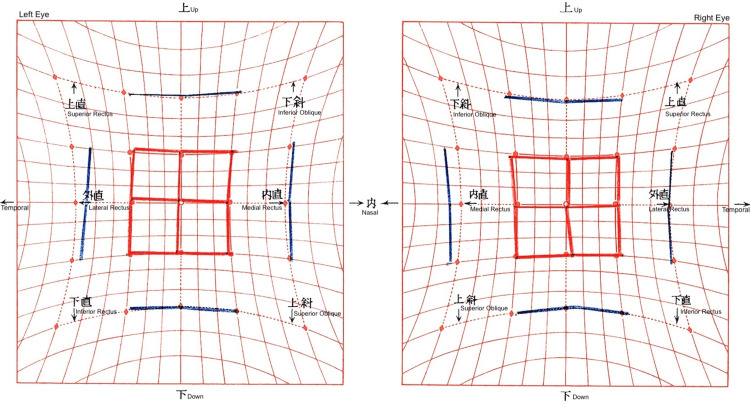
Postoperative Hess chart after left medial rectus recession The chart demonstrates excellent alignment and full ocular motility with resolution of diplopia in right gaze one month postoperatively. Although mild incomitancy persisted in left gaze, the patient remained subjectively free of diplopia

A 42-year-old male with a history of intermittent exotropia underwent bilateral lateral rectus recession and MR resection on the right eye for binocular diplopia. Although ocular alignment in primary gaze improved, residual diplopia was still noted on right gaze (10 PD esotropia) which impacted his daily activities postoperatively (Figures 9, 10). A subsequent 3.5 mm MR recession in the left eye resolved the diplopia in right gaze and restored comfortable binocular vision one month postoperatively (Figure 11).

## Discussion

Diplopia confined to secondary gaze position is often considered clinically tolerable and may be managed non-surgically [3]. However, our cases demonstrate that persistent diplopia in secondary gaze can significantly impair visual function and quality of life. Additional surgical intervention, based on precise motility assessment and tailored to individual gaze deficits, resulted in resolution or significant reduction of diplopia in the secondary gaze position and led to substantial symptomatic relief. Importantly, confirmation of incomitance using Hess chart plotting was critical in guiding surgical planning, ensuring that the residual deviation in secondary gaze was well-characterized and not misattributed to a comitant strabismus pattern.

In all three cases, MR recession was associated with improvement or resolution of residual esodeviation diplopia in secondary gaze. This finding suggests that allowing for a slight postoperative exodeviation may improve tolerance in secondary gaze and should be considered during surgical planning. Consistent with previous reports, our findings suggest that MR recession can improve or eliminate residual diplopia in secondary gaze. This outcome reflects the broader principle that the aim of adult strabismus surgery is not merely to restore primary position alignment but to expand the diplopia-free field and address incomitance across gaze positions [6,7]. Several authors have emphasized that even a small postoperative esotropia may be poorly tolerated in adults, often resulting in persistent diplopia [8,9]. Kushner has similarly noted that postoperative diplopia remains a risk when surgical goals are not carefully aligned with functional needs [10]. To mitigate this, a slight undercorrection - manifesting as a small postoperative exotropia - has been recommended to improve binocular comfort and tolerance across secondary gazes [11,12]. Our cases support this approach, highlighting the functional benefits of planning for a mild exotropic outcome during surgical decision-making.

These findings underscore the importance of a patient-centered approach to strabismus management. Even diplopia confined to secondary gaze can have functional and psychological consequences and should be addressed when it negatively impacts daily life. While primary gaze alignment remains the principal surgical goal, additional procedures may be warranted in cases where secondary gaze diplopia is symptomatic and functionally limiting. Confirmation of incomitance with Hess chart analysis provides valuable objective evidence to guide such decisions.

## Conclusions

Diplopia confined to secondary gaze is not typically considered an indication for strabismus surgery when alignment in primary gaze is satisfactory. However, in select patients whose symptoms interfere with daily function, additional surgery targeting secondary gaze deviations may be beneficial. Accurate ocular motility assessment, including confirmation of incomitance with Hess chart analysis, and individualized evaluation of visual needs are essential for optimal management.

## References

[1] (2025). Pediatric ophthalmology and strabismus. Section 6. Basic and Clinical Science Course.

[2] Takahashi S, Goseki T, Noda S, Kawanobe T, Ishikawa E, Tanaka Y, Kozawa T (2025). Reliability and validity of pre- and post-operative health-related quality of life in strabismus patients using the Japanese version of the adult strabismus questionnaire (AS-20). Jpn J Ophthalmol.

[3] Kushner BJ (1995). Management of diplopia limited to down gaze. Arch Ophthalmol.

[4] Roper-Hall G (2006). The Hess screen test. Am Orthopt J.

[5] Goseki T, Kunimi K, Shioya N, Iijima Y, Sebe M, Hosoya K, Fukaya K (2022). New device for taking nine-directional ocular photographs: "9Gaze" application. J Eye Mov Res.

[6] Howard MA (2025). Considerations in surgical correction of adult strabismus. https://www.aao.org/education/disease-review/considerations-in-surgical-correction-of-adult-str.

[7] Nihalani BR, Hunter DG (2011). Adjustable suture strabismus surgery. Eye (Lond).

[8] Hatt SR, Leske DA, Kirgis PA, Bradley EA, Holmes JM (2007). The effects of strabismus on quality of life in adults. Am J Ophthalmol.

[9] Repka MX, Lum F, Burugapalli B (2018). Strabismus, strabismus surgery, and reoperation rate in the United States: analysis from the IRIS Registry. Ophthalmology.

[10] Kushner BJ (2002). Intractable diplopia after strabismus surgery in adults. Arch Ophthalmol.

[11] Lekskul A, Supakitvilekarn T, Padungkiatsagul T (2018). Outcomes of undercorrection in surgical management and binocular vision gained of adult intermittent exotropia. Clin Ophthalmol.

[12] Kopmann S, Grenzebach U, Ehrt O, Biermann J (2024). Effectiveness of strabismus surgery in intermittent exotropia and factors influencing outcome. J Clin Med.

